# IDENTIFICATION AND INVESTIGATION OF SENESCENT CELLS IN SKELETAL MUSCLE AGING

**DOI:** 10.1093/geroni/igac059.1746

**Published:** 2022-12-20

**Authors:** Xu Zhang, Leena Habiballa, Zaira Aversa, Yan Er Ng, Joao Passos, Nathan LeBrasseur

**Affiliations:** Mayo Clinic, Rochester, Minnesota, United States; Mayo Clinic, Rochester, Minnesota, United States; Mayo Clinic, Rochester, Minnesota, United States; Mayo Clinic, Rochester, Minnesota, United States; Mayo Clinic, Rochester, Minnesota, United States; Mayo Clinic, Rochester, Minnesota, United States

## Abstract

Skeletal muscle aging is marked by the loss and atrophy of resident fibers, and the accumulation of functionally diverse cell types including fibroblasts, adipocytes, and immune cells. Senescent cells amass in multiple tissues with advancing age where they contribute to aging, chronic disease, and physical decline. The role of senescence in mediating muscle aging has become a popular and sometimes contentious topic. However, to date, this concept has not been methodically tested. In this study, we characterized the changes in cell abundance and, importantly, cell-specific transcriptional profiles with skeletal muscle aging using scRNAseq. Interestingly, we identified a small population of p16 positive fibro-adipogenic progenitors (FAPs) which, upon further investigation using immunohistochemical methods, were found to express other senescence markers. This subpopulation of FAPs did not exhibit elevation in p21 levels with age. Instead, terminally differentiated myofibers were the source of the p21 increase. Myofibers with high p21 expression exhibit a strong inflammatory phenotype, which includes activated p53 signaling pathways together with strong cytokine-cytokine receptor interactions. We further identified large amounts of cross-talk between different cell types, suggesting that senescent FAPs and myofibers could contribute to skeletal muscle aging in a paracrine manner. Importantly, these observations in mice were confirmed in human samples, suggesting the strong translational power of these findings.

